# Perceived Ageism During the Covid-19-Crisis Is Longitudinally Related to Subjective Perceptions of Aging

**DOI:** 10.3389/fpubh.2021.679711

**Published:** 2021-07-13

**Authors:** Anna E. Kornadt, Isabelle Albert, Martine Hoffmann, Elke Murdock, Josepha Nell

**Affiliations:** ^1^Department of Behavioural and Cognitive Sciences, University of Luxembourg, Esch-sur-Alzette, Luxembourg; ^2^RBS Center fir Altersfroen, Itzig, Luxembourg

**Keywords:** ageism and age-based discrimination, COVID-19, subjective age, self-perceptions of aging, older adults (50 years and above)

## Abstract

Ageism in media and society has increased sharply during the Covid-19-crisis, with expected negative consequences for the health and well-being of older adults. The current study investigates whether perceived ageism during the crisis longitudinally affects how people perceive their own aging. In June 2020, *N* = 611 older adults from Luxembourg [aged 60–98 years, *M*_*age*_(*SD*) = 69.92(6.97)] participated in a survey on their perception of the crisis. In October 2020, *N* = 523 participated in a second measurement occasion. Participants reported on perceived ageism during the crisis in different domains, their self-perceptions of aging and subjective age. In latent longitudinal regression models, we predicted views on aging at T2 with perceived ageism at T1, while controlling for baseline views on aging and covariates. Perceived ageism at T1 increased self-perceptions of aging as social loss and yielded a trend for physical decline, while there were no effects on subjective age and self-perceptions of aging as continued growth. Views on aging are powerful predictors of well-being and health outcomes in later life. Our data suggest that being the target of ageism during the crisis negatively affects older adults' self-perceptions of aging and this impact may be felt beyond the current crisis.

## Introduction

During the Covid-19 pandemic, being of higher age places people at higher risk for intensive care treatment and mortality [as do other risk factors, such as obesity or being male, (1)] when infected with Sars-Cov-2. Thus, protecting the most vulnerable members of society was rightly put at the forefront of the fight against the pandemic, and the decision which measures were implemented was often based on considerations how to best protect those with the highest risk, such as older people. However, the undifferentiated way in which especially the role of age as a risk factor was discussed and the inclusion of all people above the age of 65 into one homogeneous risk group, often neglected the multidimensionality of aging, the diversity of older people and their characteristics and thus drew criticism for fueling ageism in society [e.g., (1–3)].

Ageism, which is defined as “*stereotypes, prejudice, or discrimination against (but also in favor of) people because of their chronological age*” (4), can be displayed at different levels, within individuals, organizations, and cultures. It can take different forms, such as for example benevolent ageism (e.g., offering and insisting on unwanted help) but also more hostile forms, such as refusing older people healthcare because of their age [e.g., (5, 6)]. What both forms have in common is that older age is seen as a state of deterioration and loss of functioning (4) and that individuals from the group of older persons are all treated as members of this group, regardless of their personal characteristics. Ageism comes with massive costs for health care systems and economies [e.g., for the US, (7)], but also negative impact for the individual, for example resulting in lowered health and well-being [e.g., (8)]. Notably, not only the experience of objective instances of ageism, but also the perception thereof represents a risk for the positive development of older people [cf. (9)].

Examples for ageist discourse and actions during the pandemic are manifold. “Boomer remover,” “The old ones spoil the statistics,” “Stay home, save grandma”—these are just some of the phrases that have been used in public discourse when it comes to the description of the Sars-CoV-2 virus with relation to older adults. Numerous commentaries [e.g., (10–13)] have observed a considerable increase in ageism during the pandemic, ranging from outright discrimination, such as the decision not to provide life-saving treatment on the basis of chronological age [e.g., (14)] to more subtle, well-meant, but also impactful forms of patronizing such as strongly advising older people to self-isolate indefinitely, regardless of health status (2, 15). Cohn-Schwartz and Ayalon (16) have classified these manifestations of ageism as the “vulnerability narrative” and the “burden narrative”: Older people are homogeneously described as weak and in need to be protected from the consequences of the pandemic at any cost. At the same time, there were discussions how the protection of this supposedly most vulnerable population placed a strain on younger people, who, despite their statistically lower likeliness of having a severe course of the disease, have to adhere to strict guidelines, relinquishing their freedom and liberties for the sake of the older ones.

The instances of ageism which emerged during the crisis might have severe consequences above and beyond the current pandemic. Besides the longstanding evidence for the detrimental influence of ageism on the individual and society (7, 8), first cross-sectional studies show that perceived ageism during the pandemic is linked to increased anxiety (16) and lower well-being and subjective health of older adults (9). To possibly counteract this negative impact, it is of utmost importance to understand the consequences of ageism during the Covid-19 pandemic, and also the mechanisms through which it affects older adults' development.

Of central interest here are subjective perceptions of aging. Subjective aging refers to individuals' conceptions about their own age and aging, including self-perceptions of aging as well as how old people feel, i.e., their subjective age (17). These variables have a large impact on indicators of successful development in later life, such as cognitive, mental, and physical health, social integration, well-being and mortality [(17); for overviews, see (18)]. First empirical evidence suggests that self-perceptions of aging indeed impact well-being in older adults during the Covid-19 crisis (19).

Subjective perceptions of aging are thought to develop early in life, and while they are overall surprisingly stable, can change over the life span as a function of experiences, such as health events (20), the availability of personal resources, such as self-esteem (21), or daily stressor exposure (22). They can also change as a response to perceived ageism. Stephan et al. (23) showed that in a large sample of US older adults, perceived ageism increased participants' subjective age over time. They offered the explanation that encounters of negative social cues related to one's own age and being seen as part of the older age group can lead to stereotype assimilation, and thus to an increased subjective age. Likewise, Hooker et al. (24) found that perceived ageism decreased positive and increased negative self-perceptions of aging 4 years later, and this pathway mediated the influence of perceived ageism on health behaviors over time. Similar findings were reported with regard to depression (25). These findings are especially relevant in the current situation: Subjective perceptions of aging can change as a function of experience. Being the target of unfair treatment due to one's age, and the persistent derogatory, stereotypical portrayal (and simplified treatment) of one's age group in public discourse might have a negative impact on how people perceive their own aging in general, and the expectations they have for their future [cf. (26)].

Given the importance of subjective perceptions of aging for developmental outcomes, and their relation to perceived ageism, which has increased during the pandemic [e.g., (11, 13)], the current study set out to test whether the perception of ageism during the first months of the Covid-19 crisis affected people's self-perceptions of aging and their subjective age 3 months later. Given the multidimensional nature of subjective age (18), we were interested in self-perceptions of aging that referred to different functional dimensions (perception of aging as continued growth, physical decline, and social loss) as well as subjective age (how old people feel compared to their chronological age). Those indicators are among the most widely used in subjective aging research, and while the different dimensions have empirical and theoretical overlap, they nevertheless represent different and distinguishable facets of how people perceive their age [e.g., (27)]. Given the content of ageist messages during the pandemic within the vulnerability and burden narrative, we hypothesized that more perceived ageism should increase detrimental self-perceptions of aging as physical decline and social loss and also lead to a higher subjective age, as well as a decrease in perceptions of aging as continued growth.

## Materials and Methods

### Sample and Procedure

In June 2020, *N* = 611 community-dwelling participants from Luxembourg, aged 60–98 (*Mage* = 69.92, *SD* = 6.97) were recruited by a survey research institute (TNS ILRES). The sample was stratified for gender, age group (60–69, 70+), and residential area. 49.6% of the sample were female and 29.5% reported at least some tertiary education. The survey was carried out either by phone, for which participants were recruited via random digit dialing (*n* = 240, response rate 27%), or online, recruited from a large database of Luxembourgish residents who agreed to be contacted for online surveys (*n* = 371, response rate 40%). Participants answered questions concerning socio-demographic information, the perception of the Covid-19-crisis in Luxembourg in general, their personal situation in the crisis, perceived ageism, subjective aging, and a number of other risk and resilience factors. In October 2020, participants were invited for a second measurement occasion, in which *N* = 523 (86%) persons participated. Participants who dropped out of the study had less positive self-perceptions of aging as continued growth (see below), *t*_(604)_ = −3.64, *p* < 0.001, and lower education *t*_(598)_ = 2.39, *p* = 0.02. There were no significant differences in any other variable included in this study (all |t| < 1.70, all p >0.093). The study was approved by the Ethics Review Panel of the University of Luxembourg (ERP 20-042-C CRISIS).

### Measures

Perceived ageism at time-point 1 was assessed by asking people “During the Covid-19 pandemic, have you felt that you were treated unfairly *due to your age* in the following domains”: (1) media coverage (2) health care (3) activities of daily life (e.g., shopping) (4) within my social network (friends, family) (5) work context. Items were developed for the current study, because of their relevance in previous research on ageism [e.g., (5)] and due to their relevance in the context of the pandemic. Participants had to indicate whether they felt unfairly treated very strongly, strongly, somewhat or not at all and a latent indicator for perceived ageism was computed from all items.

At both time-points, self-perceptions of aging were assessed with the established AgeCog scales (28) in the domains of physical decline (3 items, e.g., “Aging means to me…that my health is declining”), continued growth (three items, e.g., “…that I continue to make plans”) and social loss (four items, e.g. “…that I feel lonely more often”). Participants had to rate the items on a four-point scale from completely applies to does not apply at all. Again, a latent variable was computed for each scale from the respective indicators.

Also, at both time points, participants indicated their felt age “Aside from your actual age: How old do you feel, in years?” and this number was subtracted from their chronological age, with more negative values indicating feeling younger. According to conventions, values three standard deviations above and below the mean were removed (T1: more than 38 years younger or more than 18 years older, 1.3% of cases; T2: more than 37 years younger or more than 19 years older, 0.8% of cases).

In addition, participants reported on their chronological age, gender (1 = male, 2 = female), education (with higher values indicating higher qualification), and subjective health (“How would you rate your current state of health?,” five-point scale, ranging from very good to very bad).

### Analyses

**Figure 1:**
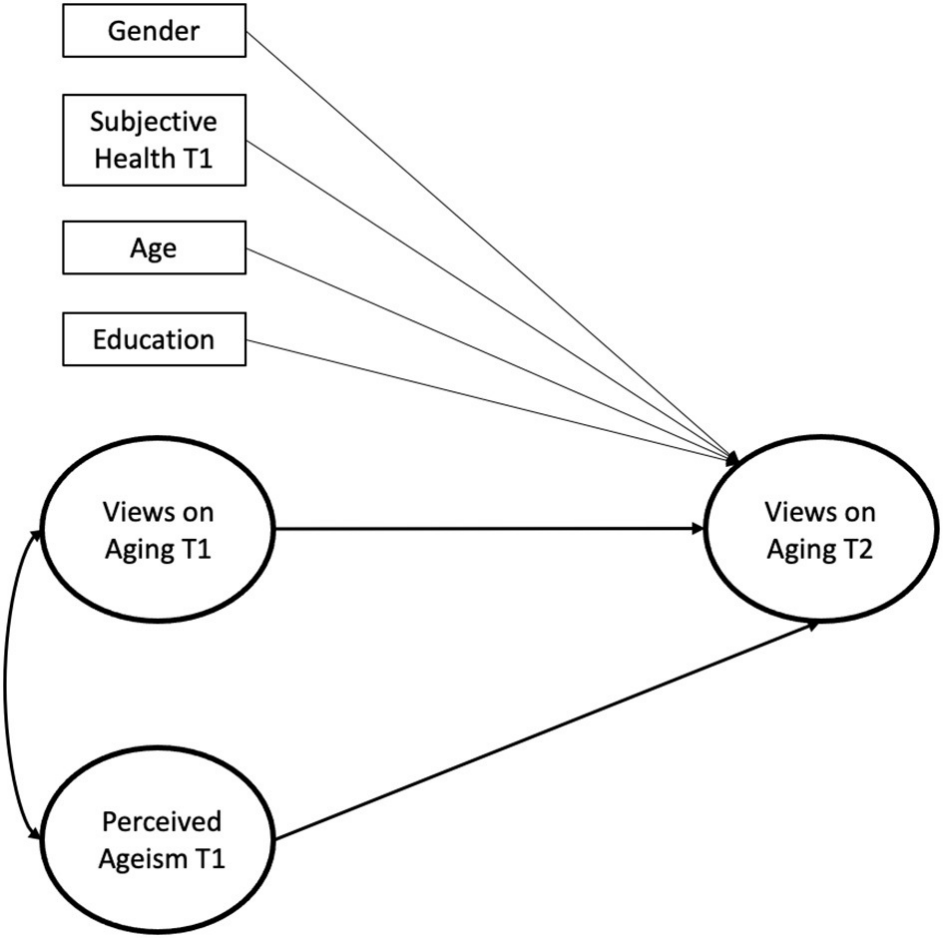
Structural equation model with covariates predicting views on aging at the second measurement occasion by perceived ageism at the first measurement occasion. For reasons of parsimony, bivariate correlations between covariates and latent variables are omitted from the figure. Corresponding factor loadings for the AgeCog scales were constrained to be equal across timepoints and error variances for the same manifest indicators were allowed to covary across time. T1, Timepoint 1; T2, Timepoint 2.

All variables were recoded so that higher values corresponded to higher endorsement. We first computed descriptive statistics and correlations for the manifest variables with SPSS 26 to address means and bivariate relationships. To address the impact of perceived ageism on views on aging, we computed latent longitudinal regression models (Figure 1) in Mplus 8 with full information maximum likelihood (FIML) estimation, one for each subjective aging indicator (physical decline, continued growth, social loss, subjective age, total four models). The respective subjective aging indicator at T2 was predicted by perceived ageism at T1, controlling for the respective indicator at T1, and, in a second model also for age, gender, education, and subjective health at T1. For the AgeCog scales, corresponding factor loadings were constrained to be equal across time-points and error variances for the same manifest indicators were allowed to covary across time.

## Results

Descriptive statistics and bivariate correlations for all variables are presented in Table 1. Perceived ageism was significantly related to self-perceptions of aging as social loss and physical decline, whereas there was no relation to continued growth and subjective age. This was mirrored in the results of the latent longitudinal analyses (Table 2): More perceived ageism at the first time-point was related to an increase in self-perceptions of aging as social loss (longitudinal effect β = 0.15). This effect remained stable, even when controlling for age, gender, and self-rated health (β = 0.16). Ageism was also significantly related to an increase in self-perceptions of aging as physical decline (β = 0.10) in a model with no covariates. However, β dropped to 0.08 and p increased to 0.10 when entering the covariates (mostly driven by the impact of subjective health). No longitudinal effects were found for self-perceptions of aging as continued growth and subjective age^1^.

**Table 1:** Descriptive statistics and manifest bivariate correlations for all study variables.

**Variable**	***n***	***M***	***SD***	**1**	**2**	**3**	**4**	**5**	**6**	**7**	**8**	**9**	**10**	**11**	**12**	**13**
1. Age	608	69.92	6.97	−												
2. PA T1	609	1.26	0.47	−0.03	−											
3. Social Loss T1	611	1.73	0.61	0.10^*^	0.28^*^	−										
4. Continued growth T1	609	2.90	0.66	−0.23^*^	−0.07	−0.30^*^	−									
5. Physical decline T1	611	2.53	0.74	0.13^*^	0.11^*^	0.39^*^	−0.29^*^	−								
6. Subjective age T1	532	−10.03	7.59	−0.10^*^	0.06	0.12^*^	−0.22^*^	0.26^*^	−							
7. Social loss T2	523	1.75	0.62	0.11^*^	0.32^*^	0.58^*^	−0.31^*^	0.29^*^	0.16^*^	−						
8. Continued growth T2	521	2.84	0.66	−0.23^*^	−0.04	−0.26^*^	0.55^*^	−0.30^*^	−0.18^*^	−0.30^*^	−					
9. Physical decline T2	522	2.47	0.73	0.09^*^	0.15^*^	0.29^*^	−0.34^*^	0.52^*^	0.22^*^	0.38^*^	−0.25^*^	−				
10. Subjective age T2	503	−8.53	7.27	−0.07	0.04	0.11^*^	−0.17^*^	0.23^*^	0.60^*^	0.14^*^	−0.16^*^	0.26^*^	−			
11. Subjective health T1	609	4.03	0.73	−0.13^*^	−0.14^*^	−0.28^*^	0.27^*^	−0.50^*^	−0.26^*^	−0.21^*^	0.21^*^	−0.42^*^	−0.21^*^	−		
12. Education	544	3.04	1.15	−0.13^*^	0.11^*^	−0.07	0.14^*^	−0.10^*^	0.05	−0.06	0.10^*^	0.01	0.10^*^	0.17^*^	−	
13. Gender	610			−0.06	−0.06	−0.03	0.01	−0.01	0.01	−0.02	0.10^*^	−0.04	−0.06	0.01	−0.16^*^	−

*PA, perceived ageism; T1, timepoint 1; T2, timepoint 2; Gender 1, male; 2, female*.

* *p < 0.05*.

**Table 2:** Model fits and standardized estimates of latent longitudinal structural equation models predicting views on aging at the second measurement occasion by perceived ageism at the first measurement occasion.

	**χ2 (df)**	***p***	**RMSEA [90% CI]**	**CFI**	**SRMR**	**Initial correlation**	**Stability**	**Longitudinal effect**
						**r_**pa1 voa1**_**	**VoA1 → VoA2**	**PA1 → VOA2**
**Model 1 (no covariates)**								
Subjective age	17.683 (13)	0.17	0.02 [0.02,0.05]	0.99	0.02	0.04	0.62^*^	0.02
Continued growth	59.361 (40)	0.02	0.03 [0.01,0.04]	0.99	0.04	−0.13^*^	0.69^*^	0.00
Social loss	203.005 (61)	< 0.001	0.06 [0.05,0.07]	0.92	0.07	0.33^*^	0.66^*^	0.15^*^
Physical decline	37.465 (40)	0.59	0.00 [0.00,0.03]	1.00	0.03	0.12^*^	0.60^*^	0.10^*^
**Model 2 (with covariates)**								
Subjective age	32.778 (29)	0.28	0.02 [0.00,0.04]	0.99	0.03	0.05	0.61^*^	0.00
Continued growth	133.974 (72)	≤ 0.001	0.04 [0.03,0.05]	0.96	0.04	−0.12^*^	0.66^*^	0.01
Social loss	310.053 (101)	≤ 0.001	0.06 [0.05,0.07]	0.90	0.06	0.33^*^	0.64^*^	0.16^*^
Physical decline	90.678 (72)	0.07	0.02 [0.00,0.03]	0.99	0.03	0.12^*^	0.51^*^	0.08^+^

*Estimates are based on maximum likelihood estimators. Models with covariates include age, gender, education and self-rated health at timepoint 1. RMSEA, root-mean-square-error of approximation; CFI, comparative fit index; SRMR, standardized root mean square residual; PA1, perceived ageism at timepoint 1; VOA1, views on aging at timepoint 1; VOA2, views on aging at timepoint 2*.

* *p < 0.05*,

+ *p =0.10*.

## Discussion

Ageism in society has strongly increased during the Covid-19 pandemic [e.g., (13)]. We set out to investigate whether the perception of such ageism influences how older people see their own aging. Our results show a differentiated picture. While there are no effects of perceived ageism in June on self-perceptions of aging as continued growth and subjective age, in October, perceived ageism was related to increased self-perceptions of aging as social loss and physical decline. The former effect is maintained when controlling for age, gender, education and subjective health. The AgeCog scale concerning social loss captures expectations of loss of respect, boredom, and loneliness. Our data show that the “vulnerability discourse,” i.e., the persistent, often patronizing advice to older people regarding the need to self-isolate, irrespective of the possible costs, might have affected older people's general expectations regarding their social development in later life. This discourse has been especially pronounced during the pandemic, indicating a possible specific historical influence pertaining to views on aging. Given the strong impact of views on aging on health and well-being in later life [e.g., (17)], as well as social integration (29), and the mediating role of self-perceptions in the link between perceived discrimination and physical and mental health [e.g., (25)] this might result in long-term negative consequences for older people.

We did not find effects of perceived ageism on self-perceptions of aging as continued growth and subjective age. Thus, the more productive perceptions of aging, which might also buffer against problematic developments, such as increasing social networks (29) or reducing morbidity as a function of depression (30), seem not to be related to perceived ageism in our sample. The effect of perceived ageism on self-perceptions of aging as physical decline also turned non-significant once subjective health was included in the model. Future studies should thus disentangle the relationship of subjective health, ageism and views on aging over time, with longer intervals and more waves of data, in order to clarify moderating and mediating relationships [cf. 17]. There are some indications that the relative advantage of older people in terms of greater emotional well-being in general has been preserved to a certain extent also during the pandemic [e.g., emotional experience (31); mood (32)]. This might also be related to the stability in productive perceptions of aging despite the perception of ageism.

Strengths of our study are the longitudinal design, the multidimensional assessment of views on aging and perceived ageism, and the latent modeling approach. Given that our study covered the time period from June to October, which in Luxembourg was rather a calm phase in-between two pandemic waves, our results could speak for enduring effects that might persist even if the protective measures have been lifted and discourse returns back to pre-pandemic times. As already mentioned before, a limitation is that we collected only two waves of data. Due to the dynamic nature of the pandemic and the volatility of measures and infection rates, additional measurement occasions would be advisable to follow developments and variable relations over longer periods of time. This would also allow to address changes in perceived ageism and the relation to views on aging as influenced by current developments, such as for example easing or tightening restrictions, or generational conflict in the wake of vaccination rollout. More measurement points would also enable us to investigate whether the observed change in views on aging longitudinally mediates the effects of perceived ageism on developmental outcomes [e.g., (24)], and also to explore the long-term relationship between views on aging, health outcomes and severe or traumatic historical and personal events [e.g., (20, 22, 33, 34)].

Further limitations concern methodological aspects. Even though we controlled for several socio-demographic and psychological characteristics, other potentially important variables (e.g., depression) were not available and might have biased our results. Besides, while we assessed perceived ageism in different domains, which is a strength, the questions were not used in previous studies and did not allow for the differentiation of hostile and benevolent forms of ageism (e.g., the well-meant take-over of chores, regardless of people's capacities and wishes). Benevolent ageism might negatively affect people's autonomy and self-worth on a more implicit and long-term level (35, 36) and thus both forms of ageism and their effects on people's self-perceptions of aging need to be addressed in future studies [cf. (9)].

Our results are in line with previous work on the impact of perceived ageism on views on aging (23, 24), however, two other studies need to be mentioned that found somewhat diverging results. In their study with data from the German aging survey, Voss et al. (37) did not find any effect of perceived ageism on the AgeCog scales, but self-perceptions of aging rather predicted perceived ageism over time. Armenta et al. (38) found that when facing ageism, people decreased their subjective age to distance themselves from their age group, whereas we did not find any effect of perceived ageism on subjective age. The divergence of results in different samples and designs speaks for the complexity of effects, which might also impact and cancel each other out, depending on the circumstances. Another interesting aspect in that regard could also be that age categories were and are very present in public discourse during the pandemic, so that the possibility to distance oneself from one's chronological age might have fluctuated^2^. These issues need to be addressed in future research. However, we are confident that our results depict the special circumstances of the Covid-19 pandemic, where ageism in media and society has increased sharply as noted above. Ageism appears more pronounced throughout society, affecting how people perceive the social aspects of their aging, with possible negative consequences for their social integration and well-being [cf. (39)]. Together with the negative effects of social isolation on older people that can be observed already [e.g., (40)], this might pose a threat to post-pandemic development which needs to be monitored. Raising awareness on the nature and consequences of ageism, and taking it into account for example in media reports, non-discriminatory risk communication and intergenerational interactions might be helpful in counteracting negative developments [cf. (9, 10)].

## Data Availability Statement

The datasets presented in this study can be found in online repositories. The names of the repository/repositories and accession number(s) can be found below: https://osf.io/zfksd/?view_only=97b0961276fc4eca895e9df7a8a3ef40.

## Ethics Statement

The studies involving human participants were reviewed and approved by Ethics Review Panel of the University of Luxembourg (ERP 20-042-C CRISIS). Written informed consent for participation was not required for this study in accordance with the national legislation and the institutional requirements.

## Author Contributions

IA received funding for the CRISIS project with contributions by AK, EM, and MH. All authors were involved in planning and preparing the data collection. AK performed the analyses and wrote a first draft of the manuscript which was then commented on by all authors.

## Conflict of Interest

The authors declare that the research was conducted in the absence of any commercial or financial relationships that could be construed as a potential conflict of interest.

## Footnotes

^1^Age did not moderate any of the presented longitudinal effects, nor did the presented results change when adding the interaction between age and perceived ageism to the models.

^2^We thank an anonymous reviewer for this interesting suggestion.
